# Chemical Analysis and Properties of Arseniated Hydrogen Gas

**Published:** 1804-05-01

**Authors:** 


### *Chemical Analysis and Properties of Arseniated Hydrogen Gas, by* PROFESSOR TROMSDORFF.

THE immortal Scheele, in his essay on arsenic and arsenic acid, mentions an inflammable arseniated gaseous fluid, of which he says: "*Hinc intelligas, hunc aerem inflammabilem esse, regulumque arsenici solutum tenere.*" Scheele states, that he obtained this gas during the solution of tin in arsenic acid. The properties of this gas, as pointed out by him, are the following. Arseniated hydrogen gas is insoluble in water; it does not render lime-water turbid; mingled with atmospheric air, no diminution of bulk ensues; on bringing the flame of a taper in contact with this mixture, a loud detonation follows, and metallic arsenic is deposited. Interesting as the observations here pointed out must appear to every chemist, the object has been neglected by succeeding operators.

Proust is the only philosopher who mentions this gas: he obtained it by digesting arsenious acid and zinc, in dilute sulphuric acid; on burning the gas, he obtained sometimes arsenious, at others arsenic acid. Being persuaded that the formation and properties of this gas deserved a closer examination, I instituted a series of experiments, the results of which are as follow.

### *Methods of obtaining arseniated Hydrogen Gas.*

1. There are a variety of processes for obtaining arseniated hydrogen. It is produced by heating tin filings in liquid arsenic acid. This method is the most expensive and most tedious. During the evolution of the gas in this process, arsenic, alloyed with tin, is precipitated, and the fluid obtained, holds in solution arseniate of tin.

2. It is likewise formed by treating in a similar manner, arsenic and iron with muriatic acid.

3. Arseniated hydrogen is also produced by heating a mixture of arsenious acid, iron filings, and muriatic acid. The fluid, in this case, contains muriate of iron and muriate of arsenic.

4. Tin filings and arsenic acid yield this gas under similar circumstances.

5. Four parts of granulated zinc and one of arsenic, treated in a similar manner with sulphuric acid, previously diluted with two parts of water, afford arseniated hydrogen very readily.

The gas obtained according to either of these processes, is nearly alike, but that produced according to the last process seems to be the most perfect gas, for it contains no excess of hydrogen. When arseniated hydrogen is produced by means of zinc, arsenic, and dilute sulphuric acid, the quantity of arseniated hydrogen is less than the quantity of hydrogen which would be obtained in decomposing water in a similar manner, without the interposition of arsenic. The residue, after the evolution of the gas has ceased, contains metallic arsenic; part of the hydrogen must therefore have acted on the oxygen of the arsenic acid, in order to reduce it to the metallic state. From what has been stated, it appears that arseniated hydrogen contains arsenic in a metallic state, and not in the state of arsenious, or arsenic acid. This will become more obvious in the sequel of this paper.

### *Physical Properties of Arseniated Hydrogen Gas.*

Arseniated hydrogen is a permanent elastic aeriform invisible fluid. It is a true chemical compound. Proust asserts that it deposits arsenic: This, however, I have never been able to observe, if the gas were *pure*. It has an alliaceous fetid smell. It extinguishes burning bodies. It is not absorbable by water; but when this fluid is freed from atmospheric air, it takes up a small quantity of the gas which becomes disengaged again by mere agitation. It does not change the colour of tincture of litmus. The specific gravity of arseniated hydrogen is, at 28′ barometrical pressure = 0,5293, or, one cubic inch (old French measure)

 weighs weighs 0,2435 grains. It is therefore lighter than oxygen, nitrogen, atmospheric air, carbonic acid, nitrous gas, ammonia, and gazeous oxyde of carbon, but heavier than hydrogen and sulphurated hydrogen gases. It is absolutely fatal to animal life.

*Chemical Properties of Arseniated Hydrogen Gas.*

Arseniated hydrogen, mingled with atmospheric air, suffers no chemical change, but mere dilution. The same holds good with respect to nitrogen. When mingled with nitrous gas, a diminution of 0,02, or 0,03 takes place, which sometimes even amounts to 0,05. To ascertain the nature of this gas, I mixed two parts of arseniated hydrogen with one of nitrous gas, and gradually added oxygen, till no further diminution of bulk ensued. On presenting to this mixture a lighted taper, a loud explosion took place, accompanied with flame. Probably part of the oxygen added, remained uncombined; for a mixture of two parts of nitrous gas, and three of arseniated hydrogen, could not be inflamed by the taper; arseniated hydrogen is miscceable with hydrogen, with carbonic acid, and with ammonia in all proportions.

Into a cylinder half filled with arsenic and hydrogen, I sent up bubbles of oxygenized muriatic acid gas. The bulk of the gas was diminished, heat was evolved, and metallic arsenic was deposited in a crystalline state. On adding to the mixture an additional dose of oxygenized muriatic acid gas, white fumes appeared, and the deposited metal vanished. The same experiment was repeated successively, taking care to add no more of the latter gas, than was just sufficient to occasion the precipitation of metallic arsenic. The collected metal yielded nitrous gas, by the affusion of nitric acid, and on adding to this mixture muriatic acid, arsenic acid was produced. The arsenic deposited in the manner stated before, when laid on ignited coals, volatilized in thick white fumes, yielding arsenious acid. The precipitation of metallic arsenic must be ascribed to the decomposition of the oxygenized muriatic acid gas; the oxygen of this gas uniting with part of the hydrogen of the arseniated hydrogen, and forming water, and thus separating the arsenic. For the arsenic is capable of being oxyded by the muriatic acid. Should it be imagined, that arsenic existed in arseniated hydrogen, in the oxydized state, and that it became precipitated by the oxygenized muriatic acid robbing it of its oxigen, we suppose things analogically erroneous, for the oxygenized mariatic muriatic acid is more capable of giving out oxygen than of taking it. The experiments of Chenevix seems perhaps hostile to this assertion; but the experiments of this philosopher merely prove that the oxygenized muriatic acid is capable of combining with an additional dose of oxygen, and constituting with it a hyperoxygenized muriatic acid. This certainly cannot be the case in the present instance, as will appear more evident from what I shall state presently.

I filled a cylinder in the mercurial pneumatic trough with arseniated hydrogen, and sent up into it as expeditiously as possible, a quantity of oxygenized muriatic acid gas. The result was evolution of heat, diminution of volume, and the inner sides of the cylinder became covered with a kind of dew. A formation of water had therefore actually taken place in this experiment. Into another dry cylinder half filled over mercury with arseniated hydrogen, I introduced dry muriatic acid gas. In this case no diminution of bulk, no separation of arsenic ensued; no change at all took place. Repeating the same experiment, I introduced into the cylinder a small quantity of water; the muriatic acid gas was absorbed, and the residue was arseniated hydrogen unaltered.

Into a cylinder half filled with oxygenized muriatic acid gas, I passed gradually arseniated hydrogen in small bubbles at a time; in this case no metallic arsenic was separated, but thick white clouds appeared. On continuing the addition of arseniated hydrogen till no more white fumes appeared, metallic arsenic was deposited. It follows from this experiment, that when a *small* quantity of arseniated hydrogen is made to act upon a *large* quantity of oxygenized muriatic acid gas, part of the oxygen of the oxygenized muriatic acid gas combines, not only with the hydrogen of the arseniated gas, and forms water, but the metallic arsenic also becomes oxydised. Reasoning from this fact, we should be inclined to believe, that a mutual decomposition of both the gases could be thus effected; but this cannot be accomplished; a diminution of bulk indeed takes place to a certain extent, but the complete disappearance of both the gases cannot be effected. If the admixture of arseniatud hydrogen with this oxygenized muriatic acid gas, be continued no longer than white clouds appear, and the residue be then examined, it will be found to consist of hydrogen and oxygenized muriatic acid gases; and the mixture detonates at the approach of a taper. The oxygenized muriatic acid

 gas gas can only be separated with difficulty by long agitation, in contact with water, and it seems as if it were become less soluble in that fluid. If the separation of this gas be accomplished, the remaining arseniated hydrogen burns with a pure flame, void of alliaceous odour, and contains no vestige of arsenic, as shall be proved hereafter. From what has been stated, the following theory may be formed.

Arsenic, in combination with a certain portion of hydrogen, constitutes arseniated hydrogen gas. On presenting to this combination oxygenized muriatic acid gas, the oxygen of this gas combines with the hydrogen, which held in solution the arsenic, and the latter is separated. If more oxygenized muriatic acid be added than is necessary for this purpose, the portion of oxygenized muriatic acid gas does not act further upon the hydrogen, but merely upon the arsenic, and the latter becomes oxydised.

Hydrogen and arseniated hydrogen may be mingled without decomposing each other; the decomposition can only be effected by the contact of fire; but if we mingle hydrogen, holding in solution sulphur and oxygenized muriatic acid gas, the decomposition and formation of water is instantly effected. This is likewise the case with arseniated hydrogen gas.

Hitherto no combination of hydrogen with a metallic substance has been known; but it is highly probable, that such combinations may exist. This indeed seems to be the case in the formation of this gas on which we are treating. If this be admitted, a division of hydrogen must take place in the following manner; one part of it must unite with the oxygen of the oxygenized muriatic acid gas, to produce water; another part must fall down with the arsenic; and another portion remains combined with caloric, in the form of hydrogen gas.

Hydrothian acid gas and arseniated hydrogen do not act upon each other. To a mixture of equal parts of hydrothian acid gas, and arseniated hydrogen gas, I added gradually oxygenized muriatic acid gas; a diminution of volume instantly took place, accompanied with liberation of heat, and a deposition of yellow sulphurized arsenic (orpiment). On adding an additional quantity of gas, the precipitate acquired a beautiful orange red colour, and on continuing the addition of oxygenized muriatic acid gas, white clouds were produced, the precipitate detached itself from the sides of the vessel, and were were gradually converted into a pulverulent substance of a yellowish white colour.

The results of these experiments are obvious, and might have been expected *a priori*. But they may serve as a test to discover the presence of arseniated hydrogen, when contained in other gases.

I mingled one cubic inch of arseniated hydrogen with ten of nitrogen, and one of hydrothian acid (sulphurated hydrogen gas ;) on adding to this mixture a small quantity of oxygenized muriatic acid gas, yellow sulphurized arsenic was instantly deposited. It is not improbable, that arsenic is likewise soluble in other gases, and in this case the hydrothian acid (liquid sulphurated hydrogen,) in conjunction with oxygenized muriatic acid, would prove a useful re-agent for discovering the presence of it.

A lighted taper immersed in a phial filled with arseniated hydrogen, is instantly extinguished; at the same time that the gas burns at the orifice of the phial with a lambent white flame, diffusing a disagreeable odour, and much white fumes, which are arsenious acid. If the gas be inflamed in a phial with a small orifice, the flame descends gradually down to the bottom of the phial, which becomes lined with a coat of crystallized metallic arsenic. In this case therefore the hydrogen alone burns.

If two parts of arseniated hydrogen be mingled with three of oxygen, and a taper be presented to the mixture, an explosion takes place; no metallic arsenic is separated, but the products are arsenious acid and water: soap-bubbles, with the mixture of these gases, explode with a bluish white flame, leaving a white smoke and strong alliaceous odour. Equal parts of arseniated hydrogen and oxygen gases, fired in like manner, do not explode so loudly, but the report is accompanied with a much more vivid flame. A stream of arseniated hydrogen, issuing from a bladder fitted with a stop-cock, and set to burn in a large receiver filled with oxygen, yielded arsenic acid. The combustion in this manner is uncommonly beautiful; the gas burns with a blue flame of uncommon splendor.

Two parts of arseniated hydrogen, and one of oxygen gas, being detonated in a close vessel by means of the electric spark, left a small residuum; on repeating the experiment, the detonating tube broke during the explosion, which prevented the examination of the residue. From what has been so far related, it becomes evident that the constituent parts of arseniated hydrogen gas, are metallic arsenic and hydrogen. Were it possible to determine termine with absolute certainty, that no increase of volume took place during the solution of arsenic in hydrogen, the proportion of the constituent parts of this gas might be ascertained thus:

| | French weight and measure. |
|---|---|
| One cubic inch of hydrogen, weighs - - | 0,0353 |
| One cubic inch of arseniated hydrogen, weighs | 0,2435 |
| Deducting the former from the latter, we get | 0,2082 |

Which is the quantity of arsenic dissolved in the gas, consequently one cubic inch of arseniated hydrogen gas consists of 0,0363 hydrogen, and 0,2082 arsenic; and one cubic inch of this gas contains about $\frac{1}{4}$ grain of metallic arsenic.

### *Habitudes of Arseniated Hydrogen to Acids.*

Into a phial, containing about eight cubic inches of arseniated hydrogen, I poured a half cubic inch of concentrated nitric acid. The moment the acid came into contact with the gas, the phial was filled with dense red fumes, a white flame pervaded the vessel, and a detonation ensued.

On repeating the experiment with dilute nitric acid, no ascension took place. The residuary gas was pure hydrogen, and the water contained arsenic acid. Fuming concentrated nitrous acid therefore is capable of oxydising the arsenic contained in this gas, at the same time that the oxygen of the acid burns with the hydrogen of the gas, and produces water; whereas weak nitric acid is only capable of oxydizing the arsenic, without acting upon the hydrogen present.

Into a glass tube, furnished with a stopper at one extremity, and closed at the other, I introduced eight cubic inches of arseniated hydrogen, to which were added two cubic inches of nitro-muriatic acid. After having agitated the fluids, on opening the tube under water, a diminution of one cubic inch took place. The residuary gas was pure hydrogen. It is remarkable, that during the addition of the nitro-muriatic acid, a black powder separated, which again disappeared on agitating the tube. Nitro-muriatic acid acts therefore in the same manner upon this gas, as oxygenized muriatic acid gas. It effects first a separation of the metallic arsenic, and then oxygenizes this metal. Liquid oxygenized muriatic acid decomposes arseniated hydrogen by mere agitation; the residue is hydrogen.

Muriatic

Muriatic acid exercises very little action upon arseniated hydrogen; but merely dissolves a minute portion of it, which may be expelled again by heat. Concentrated acetic acid has no effect upon it.

Into a glass cylinder holding eight cubic inches of arseniated hydrogen, I poured one cubic inch of concentrated sulphuric acid, and then closed the tube. At the moment of the addition of the acid, the cylinder became lined with a coat of bright metallic arsenic, so as to resemble a looking-glass. On agitating the cylinder, the coating resolved itself into a brownish black powder, which, after a few days, assumed the colour of Kermes mineral. On opening the cylinder under water, a diminution of bulk ensued, and the residuary gas proved to be hydrogen. The experiment was repeated, and yielded the same results. The sulphuric acid employed in this experiment, had acquired a penetrating pungent smell, and was examined, after having been neutralized by ammonia, in the following manner:

Ammoniate of copper, on being mingled with it, acquired a greenish colour. Hydrosulphuret of ammonia instantly occasioned a copious yellow precipitate.

Water impregnated with sulphurated hydrogen gas, occasioned a similar effect. From the results of these tests it becomes obvious, that the acid consisted of sulphuric, sulphurous, and arsenic acid. In order to be certain in this respect, I mingled a few drops of liquid arsenic acid with a mixture of sulphuric and sulphureous acid, neutralized the fluid with ammonia, and submitted it to the same tests. The results of this mixture were analogous to the former. The decomposition of the arseniated hydrogen gas, is probably analogous to the decomposition of this gas, by means of oxygenized muriatic acid gas. The sulphuric acid first gives up part of its oxygen to the hydrogen of the arseniated gas, and occasions the separation of the arsenic; which, at the expence of the remaining portion of oxygen of the sulphuric acid, becomes afterwards oxygenized, and constitutes the arsenic acid.

### *Habitudes of arseniated Hydrogen Gas to Metallic Solutions.*

I caused a current of arseniated hydrogen gas to pass through a solution of ammoniate of copper. A metallic pellicle appeared on the surface of the fluid, which suffered no other change.

Into a bottle filled with arseniated hydrogen gas, I dropped a solution of muriate of tin. On agitating the solution, it it acquired a brown colour, a partial diminution of the gas ensued, but the solution of tin was not converted into an oxydized muriate of tin, which would have been the case, if the arsenic existed in the gas in an oxydized state.

Nitrate of lead, on being brought into contact with arseniated hydrogen gas, became turbid, and deposited a precipitate, which was arseniate of lead.

Nitrate of silver submitted to the action of the gas, became instantly of an intense black, and a pellicle of metallic silver collected on the surface of the fluid. The residue of the gas, which had been made to act on the oxyde of silver for some time, had all the properties of pure hydrogen.

This experiment shows, that nitrate of silver might be employed for detecting the presence of arseniated hydrogen; for as long as a minute quantity of arsenic was present, a black precipitate ensued, whereas *pure* hydrogen has no effect upon this re-agent.

I passed into a concentrated solution of nitrate of silver, a stream of arseniated hydrogen, collected the black metallic precipitate, washed and dried it. The fluid obtained in this process did not disturb the transparency, or change the colour of ammoniate of copper. Neither liquid sulphurated hydrogen, tincture of galls, nor potash, had any effect upon it. It contained therefore neither silver nor arsenic. The precipitate before obtained, acquired a metallic lustre on being saturated; laid on ignited coals, it diffused an odour of arsenic, and it yielded by fusion a button of silver. It was an arseniate of silver.

Arseniated hydrogen passed into a solution of nitro-muriate of gold, occasioned a precipitate; on the surface of the fluid appeared a pellicle of metallic gold; and the sides of the vessel, in contact with the fluid, became beautifully gilded. The fluid through which the gas had been passed, examined in the usual manner, proved to contain no vestige either of gold or arsenic. The precipitate greatly resembled charcoal dust, interspersed with minute particles of gold.

It is highly probable, that arseniated hydrogen is capable of decomposing all metallic solutions, the basis of which is either nitric, or muriatic acid, and probably other acids.

### *Habitudes of arseniated Hydrogen Gas to various other Bodies.*

Expressed oils, on being agitated for some time in contact with arseniated hydrogen, absorbed part of the gas, and acquired a deeper colour.

Alcohol

Alcohol suffers no change from arseniated hydrogen. Solution of potash, and liquid ammonia, do not absorb it.

Such are the properties of this gas, the investigation of which I shall continue as soon as my health is restored, it being so considerably injured by the unavoidable inhalation of this gas during the course of these experiments, which gives me ample reason to conclude, that the gas must be highly poisonous.

*Enfurth, Feb.* 1803.

